# Medulloblastoma: clinicopathological parameters, risk stratification, and survival analysis of immunohistochemically validated molecular subgroups

**DOI:** 10.1186/s43046-021-00060-w

**Published:** 2021-02-08

**Authors:** Asmaa Mustafa Eid, Nehal Abd El-Ghaffar Heabah

**Affiliations:** Pathology Department, Faculty of Medicine, Tanta University, Tanta, Egypt

**Keywords:** Medulloblastoma, Molecular classification, β-catenin, GAB1, Histological types, Risk stratification, Overall survival, Progression-free survival

## Abstract

**Background:**

Medulloblastoma (MB) is a heterogeneous disease, displaying distinct genetic profiles with specific molecular subgroups. This study aimed to validate MB molecular subgrouping using surrogate immunohistochemistry and associate molecular subgroups, histopathological types, and available clinicopathological parameters with overall survival (OS) and progression-free survival (PFS) of MB patients. This study included 40 MBs; immunohistochemical staining, using β-catenin and GRB2-Associated Binding Protein 1 (GAB1) antibodies, was used to classify MB cases into wingless signaling activated (WNT), sonic hedgehog (SHH), and non-WNT/SHH molecular subgroups. Nuclear morphometric analysis (for assessment of degree of anaplasia) and Kaplan-Meier survival curves were done.

**Results:**

MB cases were classified into WNT (10%), SHH (30%), and non-WNT/SHH (60%) subgroups. Histopathological types differed significantly according to tumor location (*p*< 0.001), degree of anaplasia (*p* = 0.014), molecular subgroups (*p* < 0.001), and risk stratification (*p* = 0.008). Molecular subgroups differed significantly in age distribution (*p* = 0.031), tumor location (*p*< 0.001), histopathological variants (*p* < 0.001), and risk stratification (*p* < 0.001). OS was 77.5% and 50% after 1 and 2 years, while PFS was 65% and 27.5% after 1 and 2 years, respectively. OS and PFS were associated significantly with histopathological variants (*p* < 0.001 and 0.001), molecular subgroups (*p* = 0.012 and 0.005), and risk stratification (*p* < 0.001 and < 0.001), respectively.

**Conclusions:**

Medulloblastoma classification based on molecular subgroups, together with clinicopathological indicators, mainly histopathological types; accurately risk stratifies MB patients and predicts their survival.

## Background

Medulloblastoma (MB) is the most prevalent malignant pediatric brain tumor and accounts for up to 10% of childhood brain cancers. Advances in genome-wide analysis and gene transcription revealed that medulloblastomas are heterogeneous tumors, consisting of distinct molecular subgroups; each has a unique genomic profile [wingless signaling activated (WNT), sonic hedgehog (SHH), and non-WNT/non-SHH that further includes group 3 and group 4 medulloblastomas]. This molecular classification suggests different cellular origins with variable driving mutations [1].

Molecular classification based on DNA transcription and genome analysis is expensive and difficult for routine performance, especially in developing centers. Owing to its great importance in clinical practice, incorporation of this molecular classification into routine pathologic MBs evaluation is a must. Few studies used more simple techniques as fluorescence in situ hybridization (FISH), and immunohistochemistry (IHC), as surrogate methods for molecular subgrouping. Such techniques are easily applicable and provide reliable results on routinely processed formalin-fixed paraffin-embedded (FFPE) specimens [1].

β-catenin and GAB1 antibodies can be used to classify MB into its three main molecular subgroups (WNT, SHH, and non-WNT/SHH) [2].

WNT*/*β-catenin signaling pathway regulates a wide range of vital cellular functions including cellular proliferation, differentiation, genetic stability, apoptosis, and tissue renewal. Aberrations in this pathway are implicated in several human malignancies including colonic carcinoma, breast carcinoma, adrenocortical tumor, melanoma, high-grade glioma, and MB. WNT subgroup of medulloblastoma (10-15% of MB) comprises almost classic histology, rarely large cell*/*anaplastic (LCA) phenotype, but never desmoplastic/nodular (D/N) variant [3].

GAB1 (GRB2-Associated Binding Protein 1) belongs to Gab family and is believed to be a unique marker of SHH medulloblastoma subgroup [4]. SHH medulloblastoma subgroup (28–30% of MB) includes desmoplastic nodular (D/N) morphology, a minority of classic variant, and less commonly LCA phenotype [5].

Non-WNT/SHHMB subgroup is molecularly defined by overexpression of MYC gene, and further sub-classified into group 3 and group 4. Group 3 (25–28% of all MB) is the most aggressive MBs with a grave prognosis and a high metastatic rate at diagnosis. Classic and LCA medulloblastomas are the only histological variants encountered. Group 4 (40-45% of all MB) shows a high incidence of chromosomal copy number variations. Classic histology is the most predominant, while LCA medulloblastomas are less commonly encountered in group 4 MB [6, 7].

The current work aimed to validate MB molecular subgrouping using surrogate IHC, and associate these molecular subgroups, histopathological types, and available clinicopathological parameters with overall survival (OS) and progression-free survival (PFS) of MB patients.

## Methods

This study was carried out on 40 cases of MB, diagnosed over 8 years (2010–2017) at Pathology Department, Faculty of Medicine, Tanta University, and private laboratories. This study was carried out during the period from May 2019 to August 2020. Cases were of adequate FFPE tissue blocks, complete clinical data including age at diagnosis, sex, tumor location, computerized tomography (CT) mass size, metastasis at diagnosis (M0 or M+), type of surgery, size of residual mass after surgery, received postoperative radiation and/or chemotherapy, and complete follow-up data (recurrence and/or death). The current study was conducted after obtaining the approval from research ethics committee, Faculty of Medicine, Tanta University (reference# 34152). Informed written consent was obtained from involved participation in the study.

### Methodology

#### Histopathological examination

MB cases were classified histologically into classic, desmoplastic/nodular (D/N), and large cell/anaplastic (LCA) MBs, according to the 2016 WHO classification of tumors of the central nervous system (CNS) [5].

##### Degree of anaplasia

Anaplasia in MBs was graded into a four tired scheme: no, slight, moderate, or severe anaplasia, according to four features: (a) enlarged nuclear size; (b) increased mitotic figures; (c) numerous apoptotic bodies; and (d) high pleomorphism with conspicuous nucleoli (large cell type) or pleomorphic crowded cells with frequent molding (anaplastic type). LCA variants were identified according to the presence of severe or even moderate anaplastic features even in a focal manner [8].

### Nuclear morphometric analysis for degree of anaplasia

For histomorphometric analysis, hematoxylin and eosin (H&E) stained sections were examined under a light microscope. Ten different non-overlapping randomly selected fields from each slide were examined at a magnification (× 400). The degree of anaplasia of MB cases was assessed by quantitative analysis of the histological photomicrographs for nuclear size (measured by nuclear perimeter in microns—arc length of a nuclear boundary [9], using an image analysis software (Image J; 1.52p software 32, NIH, USA)).

### Immunohistochemical analysis

For clinical purposes, Taylor et al. recommended immunohistochemical use of β-catenin, GAB1 antibodies, to classify MBs into three molecular subgroups: WNT, SHH, and non-WNT/SHH [2]. Formalin-fixed paraffin-embedded tissue blocks were cut into 5-μm sections. After processing with xylene, graded ethanol solutions, and 3% H_2_O_2_ for 10 min, antigen retrieval was performed in 0.05 M. citrate buffer (pH = 6.0) at 100 °C for 5–10 min followed by blocking in goat serum for 10 min. Deparaffinization and antigen retrieval were performed in a Dako PT Link unit. Both high and low pH EnVision TM FLEX Target Retrieval Solutions were used at 97 °C for 20 min.

Dako automated immune-stainer (Link 48) was used for immunostaining using β-catenin antibody, a mouse monoclonal antibody (clone 12F7, sc-59737, Santa Cruz Biotechnology, Inc., USA) and GAB1 antibody, a mouse monoclonal antibody (clone H-7: sc-13319, Santa Cruz Biotechnology, Inc., USA). The slides were incubated with primary antibodies for 20–30 min, following treatment with a peroxidase-blocking reagent for 5 min. Horseradish peroxidase (HRP) reagent was added for 20 min and diaminobenzidine (DAB) chromogen solution for 10 min. Meyer’s hematoxylin was applied for counterstaining.

#### Assessment of β-catenin IHC results

Nuclear β-catenin immunoreactivity in ≥ 5% of tumor cells was considered positive. Either nuclear β-catenin immunoreactivity in < 5% of tumor cells or cytoplasmic positivity were considered negative for β-catenin expression (entire β-catenin negativity is exceptional in MB) [10]. Positive control included specimens of normal colon and colonic carcinoma. Negative control was performed by replacing the primary antibody with phosphate-buffered saline (PBS).

### Assessment of GAB1 IHC results

GAB1 positivity was detected as cytoplasmic staining in ≥ 30% of tumor cells, meanwhile percentage of positive cells < 30% was regarded as negative [11]. Positive control was tonsillar tissue. Negative control was performed by replacing the primary antibody with PBS.

Synaptophysin, NeuN, and INI immunohistochemistry further confirmed the diagnosis of anaplastic MB with rhabdoid features. IHC revealed positive cytoplasmic and nuclear reactions for synaptophysin and NeuN, respectively, as well as intact nuclear expression of integrase interactor 1 (INI 1) (to exclude atypical teratoid rhabdoid tumor [AT/RT]).

### Risk stratification

Patients were classified into standard and high-risk based on age at diagnosis (> 3 or < 3 years), size of postoperative residual mass (maximum cross-sectional area *<* 1.5 and *>* 1.5 cm^2^), histology, and metastatic disease at diagnosis (M0 or M+) [12].

### Statistical analysis

Statistical analysis was performed using the IBM SPSS software package version 20.0. (Armonk, NY: IBM Corp). Data were expressed as frequencies for categorical variables, and continuous variables were expressed as mean ± SD or median and range. The Kolmogorov-Smirnov test was used to verify the normality of distribution of variables. For comparing categorical variables, chi-square (χ2) and Monte Carlo (MC) tests were applied.

Survival analyses [overall survival (OS) and progression-free survival (PFS)] were performed. OS was the time from date of diagnosis to death or the date of last follow-up. PFS was the time interval from date of surgery to the date of progression or relapse. Kaplan-Meier survival curves were done for the significant relation with OS and PFS. Kaplan-Meier survival analysis with number at risk was done using the MedCalc Statistical Software version 18.9.1 (MedCalc Software bvba, Ostend, Belgium). *P* value < 0.05 was considered statistically significant.

## Results

### Clinical characteristics

The current study included 40 MB patients. Their clinical data are summarized in Table 1.

**Table 1:** Clinical characteristics of the studied cases

	No. (%)
Age in years	
< 3 (infants)	8 (20%)
3-16 (pediatric age)	27 (67.5%)
> 16 (adults)	5 (12.5%)
Mean ± SD.	7.7 ± 6.2
Median (min.-max.)	5 (1.8-26)
Sex	
Male	22 (55%)
Female	18 (45%)
Location of the mass	
Midline	27 (67.5%)
Lateral	13 (32.5%)
Computerized tomography (CT) mass size	
< 3 cm	21 (52.5%)
> 3 cm	19 (47.5%)
Type of surgery	
Gross-total resection	6 (15%)
Near-total resection	10 (25%)
Sub-total resection	24 (60%)
Residual size after surgery	
< 1.5 cm^2^	18 (45%)
> 1.5 cm^2^	22 (55%)
Metastasis at diagnosis	
M0	21 (52.5%)
M+	19 (47.5%)
Postoperative protocol	
No therapy	3 (7.5%)
Radiation therapy	13 (32.5%)
Radiation plus chemotherapy	24 (60%)
Risk stratification	
Standard risk	18 (45%)
High risk	22 (55%)
Recurrence rate	
No	11 (27.5%)
Yes	29 (72.5%)
Death	
Survival	20 (50%)
Death	20 (50%)

### Histopathological features, nuclear morphometric analysis for degree of anaplasia, and molecular subgroups

Based on microscopic evaluation of 40 MBs, 16 cases (40%) were of LCA histology, 14 cases of classic histology (35%), and 10 cases were D/N MB (25%).

#### Morphometric analysis for degree of anaplasia

Based on combined histopathological examination and image analysis, 18 cases (45%) showed severe anaplastic features, 15 cases (37.5%) showed moderate anaplasia, and 7 cases (17.5%) showed slight anaplasia. Mean nuclear perimeter for different histopathological types was as follows: 44.529 μm for D/N, 46.996 μm for classic, and 62.237 μm for LCA phenotype.

#### Molecular subgrouping

**Fig. 1:**
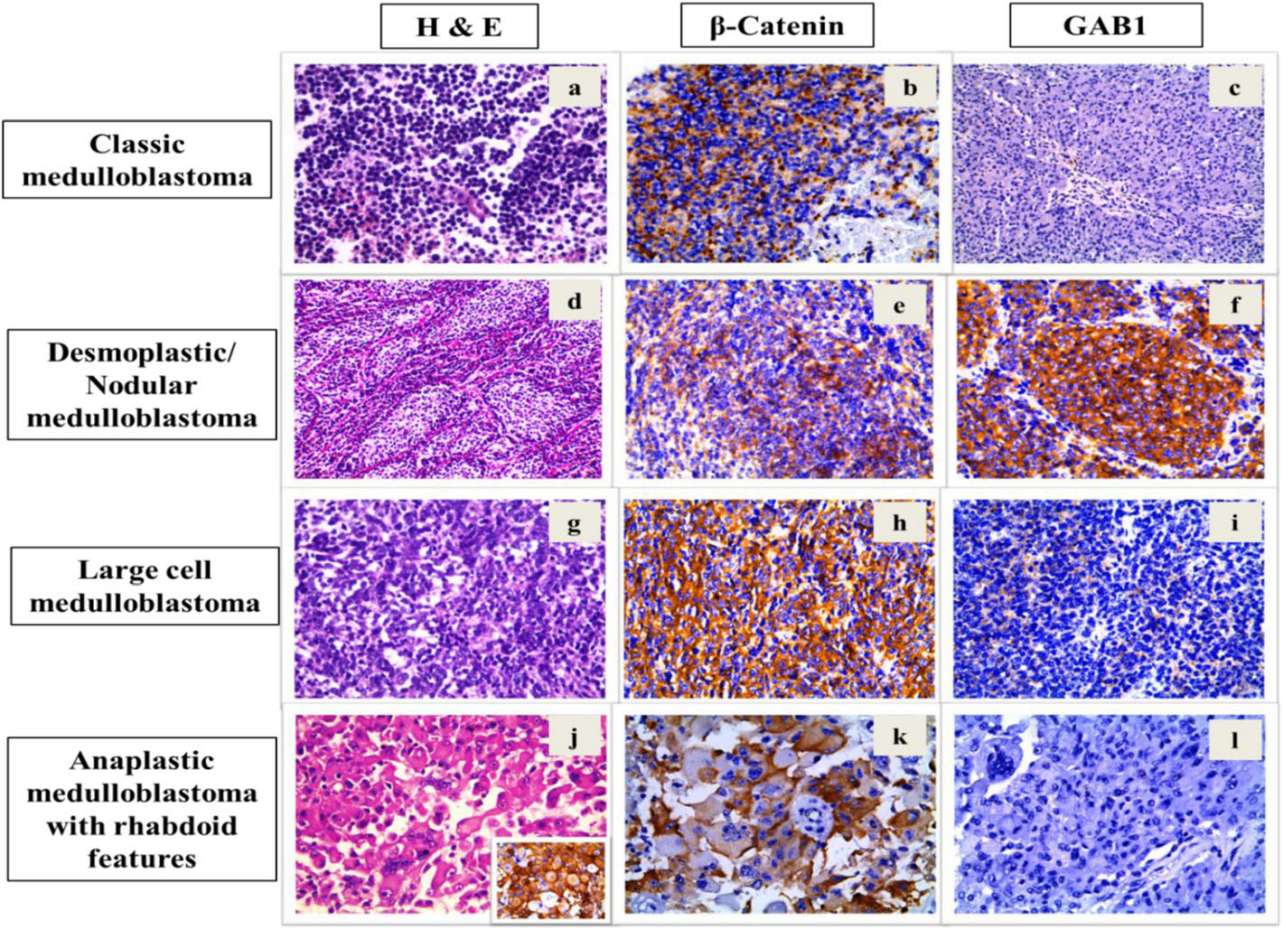
Histopathological and immunohistochemical results of MB cases. (**a**) H&E sections of classic histology (× 400), (**b**) nuclear immunoreactivity for β-catenin (× 400), (**c**) negative cytoplasmic staining for GAB1 (× 200); Both b and c characterize WNT pathway of MB, (**d**) H&E sections of D/N histology revealed nodular configuration with intervening desmoplasia (× 200), (**e**) negative nuclear expression for β-catenin (with cytoplasmic immunoreactivity) (× 400), (**f**) strong cytoplasmic staining for GAB1 (× 400); both **e** and **f** denote SHH profile, (**g**) H&E sections of large cell MB, (**h**) negative nuclear expression for β-catenin (with cytoplasmic reaction) (× 400), (**i**) negative cytoplasmic reactivity for GAB1 (× 400), (**j**) H&E sections of anaplastic MB with rhabdoid features exhibiting abundant eosinophilic glassy cytoplasm with eccentric nuclei and scattered multinucleated giant cells; confirmed by positive cytoplasmic staining for synaptophysin (inset) (× 400), (**k**) negative nuclear/positive cytoplasmic expression for β-catenin (× 400), (**l**) negative cytoplasmic reaction for GAB1 (× 400); **h**, **i**, **k**, and **l** characterize non-WNT/SHH subgroup of MB

Based on IHC staining results, the WNT subgroup (nuclear β-catenin positivity, cytoplasmic GAB1 negativity) represented 10% of cases; SHH subgroup (nuclear β-catenin negativity, cytoplasmic GAB1 positivity) represented 30% of cases, and non-WNT/SHH (both nuclear β-catenin, cytoplasmic GAB1 negativity) represented 60% of cases (Table 2, Fig. 1).

**Table 2:** Histopathological, IHC results, and molecular subgroups

	No. (%)
Histopathological types	
Classic medulloblastoma	14 (35%)
Desmoplastic/nodular medulloblastoma	10 (25%)
Large cell/anaplastic medulloblastoma	16 (40%)
Degree of anaplasia	
Slight anaplasia	7 (17.5%)
Moderate anaplasia	15 (37.5%)
Severe anaplasia	18 (45%)
β-catenin expression	
Positive nuclear expression	4 (10%)
Negative both nuclear and cytoplasmic expression	4 (10%)
Cytoplasmic expression	32 (80%)
GAB1 expression	
Negative	28 (70%)
Positive	12 (30%)
Molecular subgroups	
WNT	4 (10%)
SHH	12 (30%)
Non-WNT/SHH	24 (60%)

### Relation of histopathological types to clinicopathological parameters

The histopathological types differed significantly according to tumor location (*p* value < 0.001), degree of anaplasia (*p* value = 0.014), molecular subgroups (*p* value < 0.001), and risk stratification (*p* value = 0.008) (Table 3).

**Table 3:** Relation of histopathological types to clinicopathological parameters

	Histopathological types			***P*** value
	Classic medulloblastoma (***n*** = 14)	Desmoplastic /Nodular medulloblastoma (***n*** = 10)	Large cell/anaplastic medulloblastoma (***n*** = 16)	
**Age group (years)**				
< 3 (infants)	3 (21.4%)	2 (20%)	3 (18.8%)	MC, *p* = 0.073
3-16 (pediatric age)	11 (78.6%)	4 (40%)	12 (75%)	
> 16 (adults)	0 (0%)	4 (40%)	1 (6.3%)	
**Sex**				
Male	9 (64.3%)	4 (40%)	9 (56.3%)	*p* = 0.495
Female	5 (35.7%)	6 (60%)	7 (43.8%)	
**Location of the mass**				
Midline	13 (92.9%)	1 (10%)	13 (81.3%)	MC, *p* < 0.001*
Lateral	1 (7.1%)	9 (90%)	3 (18.8%)	
**Degree of anaplasia**				
Slight anaplasia	4 (28.6%)	3 (30%)	0 (0%)	MC, *p* = 0.014*
Moderate anaplasia	7 (50%)	4 (40%)	4 (25%)	
Severe anaplasia	3 (21.4%)	3 (30%)	12 (75%)	
**Molecular subgroups**				
WNT	3 (21.4%)	0 (0%)	1 (6.3%)	MC, *p* < 0.01*
SHH	0 (0%)	10 (100%)	2 (12.5%)	
Non-WNT/SHH	11 (78.6%)	0 (0%)	13 (81.3%)	
**Risk stratification**				
Standard risk	7 (50%)	8 (80%)	3 (18.8%)	*p* < 0.008*
High risk	7 (50%)	2 (20%)	13 (81.3%)	

*MC* Monte Carlo

*Statistically significant at *p* ≤ 0.05

The majority of classic MBs and LCA were diagnosed at pediatric age (78.6% and 75%, respectively); D/N MBs were distributed among all age groups (however, no significant relation was detected between histopathological types and age of the patients). Most of classic and LCA MBs were located at the midline (92.9% and 81.3%, respectively), whereas 90% of D/N cases were located laterally at the cerebellar hemispheres.

Half of classic histology showed moderate anaplasia, while 75% of LCA cases showed marked anaplasia. Regarding the molecular subgroups, 78.6% of classic histology was of non-WNT/SHH profile, 21.4% of WNT type. D/N cases were exclusive of SHH type (100%). LCA histology showed mostly (81.3%) non-WNT/SHH profile.

Considering risk stratification, 50% and 80% of classic and D/N cases were of standard-risk group, respectively, while 81.3% of LCA cases were of high-risk group.

### Relation of molecular subgroups to clinicopathological parameters

The molecular subgroups differed significantly in age distribution (*p* value = 0.031), tumor location (*p* value < 0.001), histopathological variants (*p* value < 0.001), and risk stratification (*p* value < 0.001). No significant relation was detected between the molecular subgroups and degree of anaplasia of the studied cases (Table 4).

**Table 4:** Relation of molecular subgroups to clinicopathological parameters

	Molecular subgroups			***P*** value
	WNT (***n*** = 4)	SHH (***n*** = 12)	Non-WNT/SHH (***n*** = 24)	
**Age group (years)**				
< 3 (infants)	0 (0%)	2 (16.7%)	6 (25%)	MC, *p* = 0.031*
3-16 (pediatric age)	3 (75%)	6 (50%)	18 (75%)	
> 16 (adults)	1 (25%)	4 (33.3%)	0 (0%)	
**Sex**				
Male	1 (25%)	5 (41.7%)	16 (66.7%)	MC, *p* = 0.183
Female	3 (75%)	7 (58.3%)	8 (33.3%)	
**Location of the mass**				
Midline	4 (100%)	1 (8.3%)	22 (91.7%)	MC, *p* < 0.001*
Lateral	0 (0%)	11 (91.7%)	2 (8.3%)	
**Histopathological types**				
Classic medulloblastoma	3 (75%)	0 (0%)	11 (45.8%)	MC, *p* < 0.001*
Desmoplastic/nodular medulloblastoma	0 (0%)	10 (83.3%)	0 (0%)	
Large cell/anaplastic medulloblastoma	1 (25%)	2 (16.7%)	13 (54.2%)	
**Degree of anaplasia**				
Slight anaplasia	2 (50%)	3 (25%)	2 (8.3%)	MC, *p* = 0.255
Moderate anaplasia	1 (25%)	5 (41.7%)	9 (37.5%)	
Severe anaplasia	1 (25%)	4 (33.3%)	13 (54.2%)	
**Risk stratification**				
Standard risk	4 (100%)	10 (83.3%)	4 (16.7%)	MC, *p* < 0.001*
High risk	0 (0%)	2 (16.7%)	20 (83.3%)	

*MC* Monte Carlo

*Statistically significant at *p* ≤ 0.05

Regarding WNT tumors, 75% of WNT tumors were detected among pediatric age (3-16 years) and were not seen in infants. They were all located in the midline and were mainly of classic histology (75%). All WNT cases showed standard risk of stratification.

SHH tumors were detected among all age groups; 50% of cases were detected among 3-16 years, 33.3% of cases were diagnosed in adults (> 16 years) and 16.7% of cases were diagnosed in infants (< 3 years). Most SHH MBs were laterally located (91.7%). It included D/N (83.3%) as well as LCA (16.7%) phenotypes; 83.3% of SHH cases showed standard-risk.

Non-WNT/SHH MBs were predominantly diagnosed in the pediatric age group (75%), 22 cases (91.7%) were located at midline; LCA and classic histology (54.2%, 45.8%, respectively) were seen in this subgroup. The majority of non-WNT/SHH MBs were high-risk tumors.

### Survival analysis

**Fig. 2:**
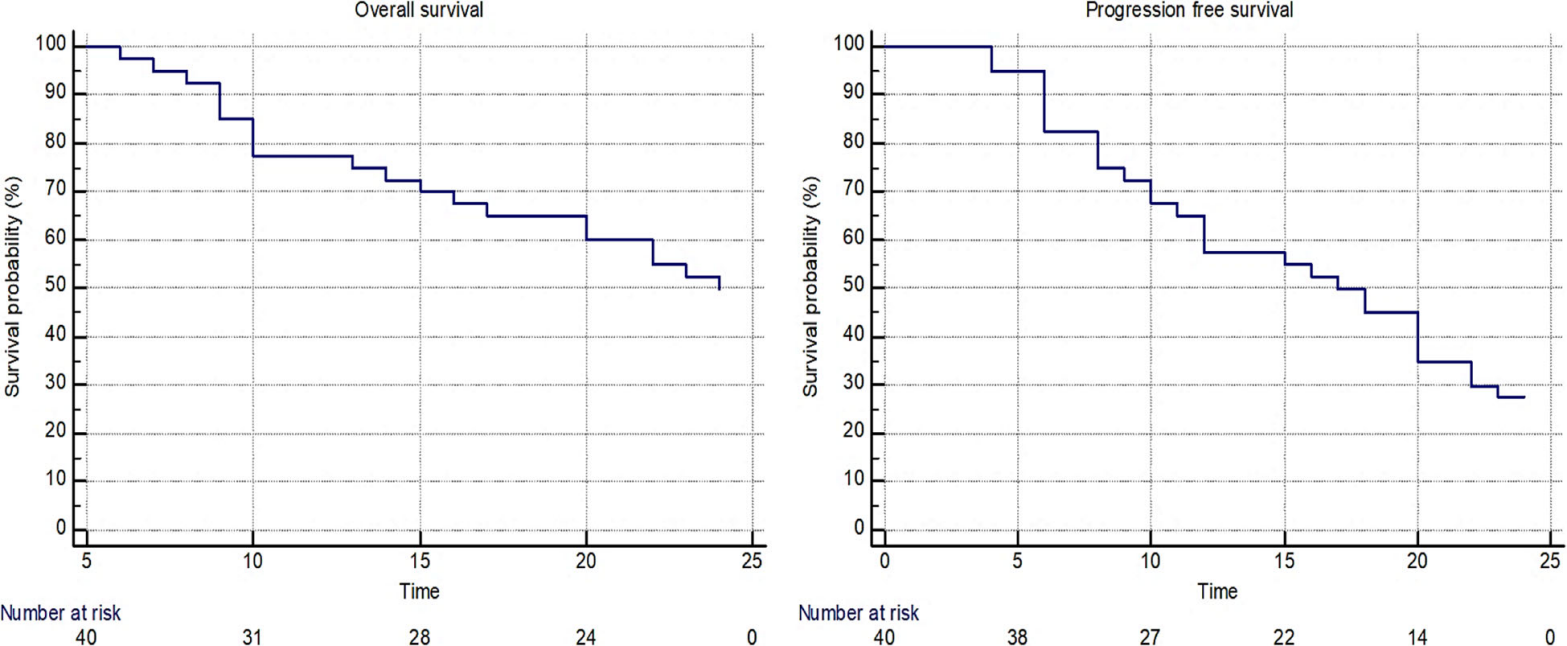
Kaplan-Meier curves for overall survival (OS) and progression-free survival (PFS) in medulloblastoma patients

The follow-up period was 2 years (24 months). The OS was 77.5% and 50% after 1 and 2 years, respectively, with a mean of 19.1 months and median of 24 months (95% CI, 17.1-21.1). The PFS was 65% and 27.5% after 1 and 2 years, respectively, with a mean of 15.83 months and median of 17 months (95% CI, 13.6-18.1) (Table 5, Fig. 2).

**Table 5:** Overall survival (OS) and progression-free survival (PFS) of the studied cases

	Mean (months)	95% CI	Median (months)	% 1 year	% 2 year (end of study)
Overall survival (OS)	19.1	17.1-21.1	24	77.5%	50%
Progression-free survival (PFS)	15.83	13.6-18.1	17	65%	27.5%

*CI* confidence interval

### Relations of OS and PFS to different clinicopathological parameters

**Fig. 3:**
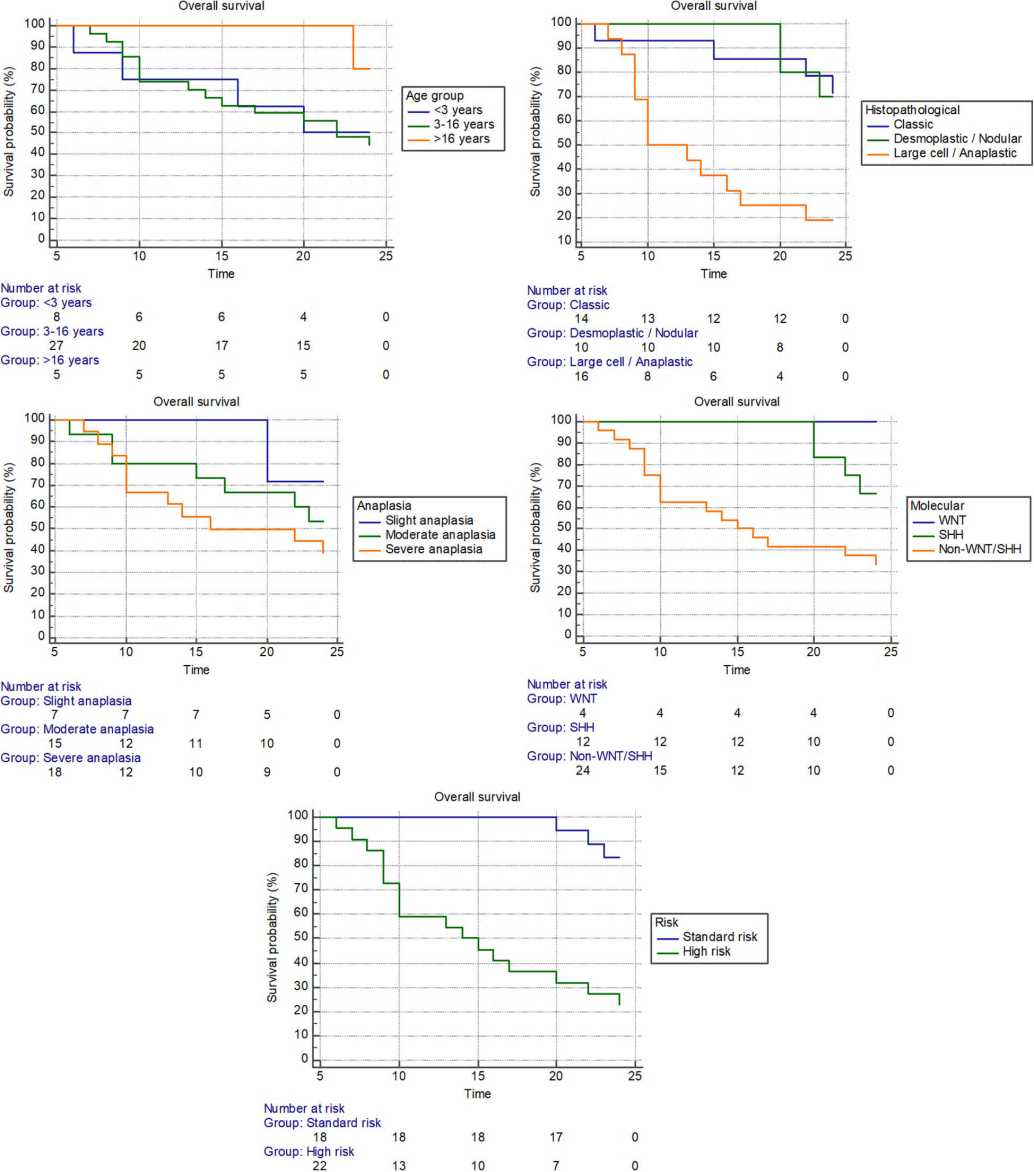
Kaplan-Meier curves for overall patients’ survival (OS), in relation to age group, histopathological types, degree of anaplasia, molecular subgroups, and risk stratification

**Fig. 4:**
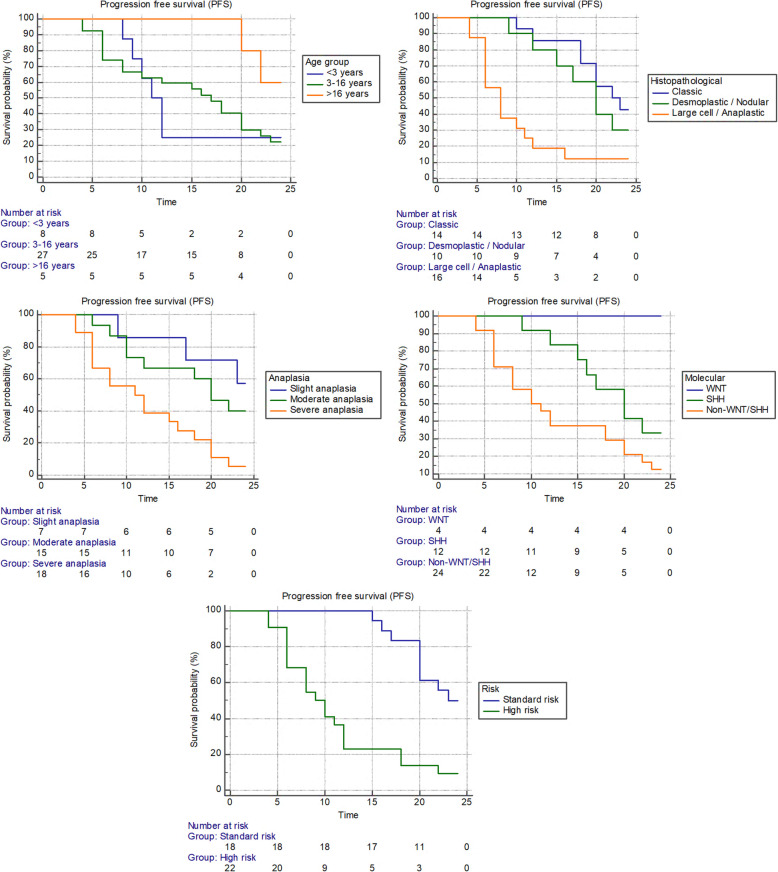
Kaplan-Meier curves for progression-free survival (PFS), in relation to age group, histopathological types, degree of anaplasia, molecular subgroups, and risk stratification

Kaplan-Meier curves revealed that both OS and PFS associated significantly with histopathological variants (*p* value < 0.001 and 0.001), molecular subgroups (*p* value = 0.012 and 0.005), and risk stratification (*p* value < 0.001 and < 0.001), respectively. MBs of LCA histology exhibited the worst OS and PFS (18.8% and 12.5%, respectively). Among the molecular subgroups, WNT had the best outcome with excellent PFS (100%), and the non-WNT/SHH showed the worst OS (33.3%). Both OS and PFS were poor with high-risk group patients (22.7% and 9.1%, respectively). Furthermore, PFS was significantly associated with the degree of cellular anaplasia, being worst with severe anaplasia (5.6%) (*p* value = 0.003) (Table 6, Figs. 3 and 4).

**Table 6:** Relation of overall survival (OS) and progression-free survival (PFS) to different clinicopathological parameters

	Overall survival (OS)				Progression-free survival (PFS)			
	Mean	Median	% End of study	***P*** value	Mean	Median	% End of study	***P*** value
**Age group (years)**								
< 3 (infants)	18.38	20	50%	0.363	13.75	11	25%	0.182
3-16 (pediatric age)	18.44	22	44.4%		15.15	17	22.2%	
> 16 (adults)	23.8		80%		22.8		60%	
**Histopathological types**								
Classic medulloblastoma	21.93^#^		71.4%	< 0.001*	20.5^#^	22	42.9%	0.001*
Desmoplastic/nodular medulloblastoma	23.1^#^		70%		18.7^#^	20	30%	
Large cell/anaplastic medulloblastoma	14.13	10	18.8%		9.94	8	12.5%	
**Degree of anaplasia**								
Slight anaplasia	22.86		71.4%	0.274	20.71^#^		57.1%	0.003*
Moderate anaplasia	19.53		53.3%		18^#^	20	40%	
Severe anaplasia	17.28	16	38.9%		12.11	11	5.6%	
**Molecular Subgroups**								
WNT	24^#^		100%	0.012*	24		100%	0.005*
SHH	23.08^#^		66.7%		18.92^#^	20	33.3%	
Non-WNT/SHH	16.29	15	33.3%		12.92^#^	10	12.5%	
**Risk stratification**								
Standard risk	23.61		83.3%	< 0.001*	21.61	23.0	50%	< 0.001*
High risk	15.41	14	22.7%		11.09	9.0	9.1%	

*OS* overall survival

^**#**^Statistically significant with large cell/anaplastic medulloblastoma

^**#**^Statistically significant with non-WNT/SHH

*PFS* progression-free survival

^**#**^Statistically significant with large cell/anaplastic medulloblastoma

^**#**^Statistically significant with severe anaplasia

^**#**^Statistically significant with WNT

## Discussion

Medulloblastoma patients, sharing the same WHO histopathological type, have peculiar genetic backgrounds and different prognoses. Therefore, diagnosis of MB requires a combined routine histopathological evaluation (including microscopic type and histological grade of malignancy “WHO Grade IV”) with additional molecular features, to give an accurate integrated (or the so-called “layered”) diagnosis, and to allow a more refined risk stratification [5].

In the current study, MB histopathological types included classic (35%), D/N (25%), and LCA (40%) of MBs. These histopathological types were significantly associated with tumor location, degree of anaplasia, molecular subgroups, and risk stratification. Most LCA cases showed marked degree anaplasia. Most of the classic and D/N cases in our results were of standard-risk group, while most of LCA cases were of high-risk group. These results were in harmony with Ellison et al., who reported that high-risk disease was associated with LCA phenotype and metastasis at diagnosis (M+) [1]. Also, Jiang et al. noted that LCA histology was an independent risk factor with a grave prognosis and claimed that such diagnoses should require intensive treatments [13].

In our results, no significant relation could be detected between the histopathological types and age of the patients. However, Al-Halabi et al. supposed that D/N medulloblastomas contributed to most MBs in infancy and adulthood, sparing the pediatric period [14].

The molecular subgroups in our study differed significantly in age distribution, tumor location, histopathological variants, and risk stratification. WNT tumors represented 10% of cases; most of them were detected among pediatric age (3-16 years) and were mainly of classic histology. Our results matched those of Ellison et al., who stated that WNT subgroup was the rarest (10% of all MBs) and that WNT medulloblastomas were almost all classic tumors (81% of their cases) and presented between the ages of 6-12 years [1]. According to Pietsch et al., WNT MBs mostly occur in children older than 3 years or teenagers [15]. Taylor et al. stated that WNT medulloblastomas rarely have LCA histology, but even with this histology, they showed an excellent prognosis [2].

In the current study, all WNT MBs located at midline, which was in agreement with Pietsch et al., who declared that WNT tumors were located mainly in the midline (their cell of origin derives from the lower rhombic lip) [15]. Gibson et al. also found that among MB cases in a mouse model study, WNT subgroup arose from the midline of the brain stem [16].

In our study, all WNT cases exhibited standard-risk stratification. Ellison et al. reported that some cases with high-risk features (including LCA morphology or M+) showed favorable outcomes, interestingly, when associated with WNT profile [1].

SHH tumors, in this study, enclosed 30% of cases. The same finding was detected by Northcott et al. and Pietsch et al., as SHH subgroup represented 30% of their MBs [5, 17]. SHH tumors in this study were detected in all age groups, mainly among pediatric age group (3-16 years). In contrast, Zhukova et al. and Pietsch et al. found that SHH tumors had a bimodal age distribution affecting both the infants and adults, sparing the pediatric period [5, 18]. Kool et al. reported that SHH medulloblastomas were found in infants and adults and occurred much less frequently in patients aged 3–15 years [19].

In the current study, most SHH MBs were laterally located. Gibson et al. reported that these tumors derived from the cerebellar granular precursor cells of the external granular layer (originated laterally from the cerebellar hemispheres) [16].

All D/N MBs in this study were of SHH type. Pietsch et al. reported that D/N variant was almost exclusive for SHH-MB, followed by classic and LCA subtypes [5]. Ellison et al. also stated that all desmoplastic tumors were included in the SHH pathway [1]. However, Taylor et al. reported that SHH medulloblastomas included both desmoplastic types and not desmoplastic/nodular types (up to 50%) [2].

Non-WNT/SHH MBs (60% of our cases), were predominantly diagnosed in the pediatric age group (3-16 years) and were located mainly at the midline. Most of its cases were LCA and classic MBs and were high-risk tumors. Cho et al. and Tamayo et al. reported that non-WNT/SHH MB constituted the most common molecular subgroup and that the MBs of this group located in the midline filling the fourth ventricle [6, 7]. According to their studies, two histologic variants are encountered in this group, classic and LCA. Also, they reported that non-WNT/SHH MBs were of high-risk group with dismal prognosis.

In this study, the 2-year OS was 50% and the 2-year PFS was 27.5%; Tarbell et al. reported a higher 5-year OS (60%) [20]. In our study, no association was detected between OS and age at diagnosis. However, Sirachainan et al. reported that the 5-year OS rate in children (3-16 years) was 60.6%, whereas children in a study by Nalita et al. had a 53.8% 5-year OS rate [21, 22]. Schwalbe et al. categorized SHH patients into two groups (SHH infant and SHH child); which had a 58 and 48% 10-year OS, respectively [4].

The OS and PFS in our study, associated significantly with histopathological types, molecular subgroups, and risk stratification. Histologically, classic and D/N types showed nearly similar OS (71.4% and 70%, respectively), with PFS of 42.9% and 30%, respectively. LCA histology exhibited the worst OS and PFS (18.8% and 12.5%, respectively). Similarly, Louis et al. reported that D/N variant exhibited the best prognosis, whereas, LCA variant had a poor prognosis [23]. Yet, Nalita et al. found no significant differences of survival rates between histological variants [22]. Gupta et al. suggested that MB histology could determine patients’ outcomes (rather than other prognostic factors such as age, concurrent CNS involvement, visceral metastases, or time to relapse) [24].

In this work, patients with severe anaplasia showed significantly worse PFS (5.6%), Giangaspero et al. also found that progression-free survival for MBs with severe anaplasia was significantly shorter than tumors with slight or moderate anaplastic features [25].

In our study, molecular subgroups were prognostically important, with significantly different survival rates. WNT tumors had the best outcome with excellent PFS, and non-WNT/SHH showed the worst and shortest OS (33.3%). SHH medulloblastomas had an intermediate (66.7%) OS. Ellison et al., Kool et al., Northcott et al., and Taylor et al. reported the best outcome and a high 5-year OS (~ 95%) for the WNT subgroup, an intermediate (75–80%) OS for SHH MBs, while the worst and shortest survival for non-WNT/SHH subgroup [1, 2, 17, 19]. Ramaswamy et al*.* also confirmed that the WNT subtype had the best clinical outcome, with a 5-year OS > 95% [26]. Cho et al*.* found that the SHH subgroup had intermediate prognosis, with a 70% 5-year OS [6].

According to Thompson et al., the prognosis of WNT MB was excellent, even in the presence of poor outcome indicators as somatic TP53 mutation, incomplete resection, and/or metastatic disease at presentation [27]. Many studies explained that the good outcome of WNT subgroup was due to the presence of WNT antagonistic secretions that modifies the permeability of blood-brain barrier; allowing high penetrance of chemotherapeutic agents into the tumor site [28]. This could permit a less aggressive approach in treating WNT tumors [3].

In combination with clinical and pathological outcome indicators, molecular markers are not only prognostically important but would also facilitate the use of targeted therapies, such as GDC-0449, a novel SHH pathway inhibitor, particularly in infants and adults [1].

In the current study, both OS and PFS were poor with high-risk group patients (22.7% and 9.1%, respectively), while in the standard-risk group, the OS and PFS were 83.3% and 50%, respectively. Nalita et al*.* also reported 84.4% and 42.8% OS rates of standard-risk and high-risk groups, respectively [22].

Tarbell et al., Ramaswamy et al., and Ramaswamy et al*.* reported higher 5-year survival rates (for high-risk MBs) reaching 60% [20, 26, 29]. Sirachainan et al*.* reported OS rates of standard-risk and high-risk groups of 58–85% and 32–70%, respectively [21]. Thompson et al*.* reported that patients with postsurgical residual tumor > 1.5 cm^2^ (an indicator of high-risk disease) had worse PFS and required aggressive treatment options [27].

Clinical trials should incorporate key molecular profiles, including subgroup information, genetic, cytogenetic, and epigenetic changes, of this diverse disease entity that can suggest precise patients’ outcomes or predict rational treatment strategies [30].

The rarity of some WHO subtypes of MB; specifically, medulloblastoma with extensive nodularity, is considered a limitation of this study. Larger studies including all the histopathological types of medulloblastoma are recommended.

## Conclusions

In conclusion, histopathological types, molecular subgroups (determined by β-catenin and GAB1 immunohistochemistry), and risk stratification are important prognosticators, and are associated with overall and progression-free survival of MB patients. Patients with the same pathological type of MB may have distinct genetic backgrounds and different prognosis. Advanced molecular testing is recommended to yield better results, confirm the current data and further classify each molecular subgroup.

## Data Availability

The datasets used and/or analyzed during the current study are available from the corresponding author on reasonable request.

## Abbreviations

CI: Confidence interval
CNS: Central nervous system
CT: Computerized tomography
D/N: Desmoplastic/nodular
DAB: Diaminobenzidine
FFPE: Formalin-fixed paraffin-embedded
FISH: Fluorescence in situ hybridization
GAB1: GRB2-Associated Binding Protein 1
H&E: Hematoxylin and eosin
HRP: Horseradish peroxidase
IHC: Immunohistochemistry
INI 1: Integrase interactor 1
LCA: Large cell*/*anaplastic
MB: Medulloblastoma
MC: Monte Carlo
OS: Overall survival
PFS: Progression-free survival
SHH: Sonic hedgehog
WHO: World Health Organization
WNT: Wingless signaling activated
*χ*2: Chi-square
